# Leveraging biofluid markers as a real‐time window to understand molecular drivers of sex differences in AD clinical trials

**DOI:** 10.1002/alz.090948

**Published:** 2025-01-09

**Authors:** Kaitlin B. Casaletto

**Affiliations:** ^1^ Memory and Aging Center, UCSF Weill Institute for Neurosciences, University of California, San Francisco, San Francisco, CA USA

## Abstract

Women account for almost two‐thirds of Alzheimer’s disease (AD) cases, yet evidence significantly less clinical benefit from recently deployed amyloid‐lowering therapies. To close this disparity gap, there is an urgent need to identify biological drivers of sex differences in the manifestation and clinical response to AD therapeutics. A recent review of multi‐omic studies of AD reported >75% of studies showed female‐specific changes at the molecular level (vs. 20% showing male‐specific changes), including enrichment in innate and adaptive immune system changes. However, the majority of these studies rely on brain tissue, which lacks temporal resolution needed to address questions related to real‐time biological responses to treatment. We propose that deep molecular phenotyping of human biofluids [blood, cerebrospinal fluid (CSF)] may offer a key window to understand in‐vivo biological signatures driving sex differences in response to AD therapies. Technological advancements in biofluid quantification now enable early, scalable detection of AD biology and have been used to show target engagement for recent amyloid antibody therapies. Both Lecanemab and Donanemab show significant improvements in CSF and plasma phosphorylated tau (p‐tau) and GFAP, for instance. Yet, despite p‐tau and GFAP evidencing sex differences in converging observational studies, clinical trials have not historically sex disaggregated their data. Newer high‐throughput platforms now allow capture of >thousands of analytes from one human biofluid sample. These approaches are shedding light on novel pathways implicated in AD, evidence cross‐tissue utility, and may allow dynamic quantification of the molecular signatures that change with AD treatment in a person‐specific manner. Human in‐vivo proteomic studies in other diseases highlight their utility for disentangling sex‐differences biology. For instance, a recent study examining the most commonly used medications in adults identified hormonal contraceptives as having the single largest effect on the plasma proteome. In cardiovascular disease, large‐scale plasma proteomics have identified differential pathways underlying cardiac disease in men versus women and identified novel molecular targets that may explain sex differences in response to heart failure treatments. In this session, we will underscore the opportunity to leverage in‐vivo biofluid analytics to develop a sex‐aware understanding of AD therapeutics.

